# Study on the Adsorption Behavior of a Cellulose Nanofibril/Tannic Acid/Polyvinyl Alcohol Aerogel for Cu(II), Cd(II), and Pb(II) Heavy Metal Ions

**DOI:** 10.3390/nano15141063

**Published:** 2025-07-09

**Authors:** Xuejin Zhang, Yulong Tian, Huanhuan Chen, Ying Liu, Shuaichuang Han, Minmin Chang, Jingshun Zhuang, Qingzhi Ma

**Affiliations:** 1 School of Environment and Natural Resources, Zhejiang University of Science and Technology, Hangzhou 310023, Chinaminminchang@zust.edu.cn (M.C.); jzhuang@zust.edu.cn (J.Z.); 2 Zhejiang Jinlong Recycle Resources Technology Co., Ltd., Quzhou 324000, China; 3 CAS Key Laboratory of Standardization and Measurement for Nanotechnology, National Center for Nanoscience and Technology, Beijing 100190, China

**Keywords:** tannic acid, nanocellulose, heavy metal ions, adsorption, kinetics, Thermodynamics

## Abstract

Nanocellulose-based composite aerogels have the advantages of high porosity, biodegradability, and biocompatibility, with wide applications in many fields, such as adsorption, separation, energy storage, and heat insulation. In this study, a nanocellulose-based composite aerogel (NCA) was prepared using the one-pot method with cellulose nanofibrils (CNFs), tannic acid (TA), and polyvinyl alcohol (PVA) as raw materials. The adsorption behaviors of Pb^2+^, Cd^2+^, and Cu^2+^ were also studied. FT-IR analysis confirmed that TA successfully solidified on the nanocellulose, while SEM analysis revealed that the prepared NCA exhibited significantly higher porosity compared with the cellulose nanofibril-only aerogel. The results of the adsorption experiment demonstrated that the adsorption behavior of heavy metal ions using the prepared NCA followed pseudo-second-order kinetics. The adsorption isotherms fit well with the Langmuir adsorption model, indicating that the process of aerogels adsorbing heavy metal ions is that of monolayer adsorption. Under conditions of pH 6 and an initial heavy metal ion concentration of 100 mg/L, the maximum adsorption capacity calculated for the prepared NCA was up to 196.850 mg/g, 181.488 mg/g, and 151.515 mg/g for Cu^2+^, Cd^2+^, and Pb^2+^, respectively. Furthermore, the prepared NCA exhibited excellent reusability, with more than 90% efficiency retained after three cycles. NCAs have the potential to become an efficient material for absorbing heavy metal ions in water.

## 1. Introduction

With the continuous development of the economy and of social life, water pollution with toxic substances has become a significant challenge. Heavy metals are defined as elements with a density greater than 5 g/cm^3^, such as lead (Pb), cadmium (Cd), and chromium (Cr) [1]. While certain heavy metal ions are essential for life, an excess can accumulate in biological tissues due to their non-biodegradable nature. These accumulated ions may interact with DNA, proteins, and enzymes, potentially leading to multiple organ failure in the lungs, liver, heart, and cardiovascular system, posing significant risks to human health [2]. Therefore, exploiting high-efficiency methods to remove heavy metal ions from water bodies remains a necessity [3].

Several approaches have been developed for removing heavy metal ions, including chemical precipitation [4], ion exchange [5], bioremediation, adsorption, and mechanical extraction [6]. Among them, adsorption is a promising method in water treatment, being simple to operate and cost-effective [7]. Aerogels have received considerable attention in water restoration due to their high porosity and specific surface area alongside a porous structure and adjustable surface chemistry. The inorganic aerogels developed, such as carbon nanotubes, graphene [8], and silicon dioxide [9], have already exhibited excellent mechanical properties and absorbability. Some disadvantages, such as non-biodegradability, high energy costs for preparation and regeneration, and secondary pollution, limit their practical application. Therefore, it is urgent to develop an environmentally friendly and cost-effective aerogel adsorbent with a high adsorption capacity and multiple functions for wastewater treatment.

Nanocellulose-based composite aerogels (NCAs) have emerged as a high-performance adsorbent in water-body purification due to their unique features, including abundant surface oxygen-containing groups, biodegradability, biocompatibility, and sustainability [10]. The modification of nanocellulose-based aerogels by their functional groups results in significant adsorption and catalytic degradation effects on heavy metal ions [11], organic dyes [12], and gas [13]. As such, their suitability for treating industrial wastewater relates to their high throughput and large adsorption capacity. NCAs are commonly used to remove heavy metals from water via adsorption and desorption processes.

The modification methods of nanocellulose include oxidation, etherification, esterification and graft copolymerization. Sun et al. prepared dicarboxylic nanocellulose with NaIO_4_ and NaClO_2_ as oxidants. Compared with traditional nanocellulose oxidized only by NaIO_4_, the dicarboxylic nanocellulose has a maximum adsorption capacity of 184.2 mg/g for copper ions because of its higher carboxyl content. NCAs are widely used to remove heavy metals from water by adsorption and desorption processes. These methods include using nanocellulose/sodium alginate/carboxymethyl-chitosan aerogel [14], covalent organic frameworks such as cellulose nano-aerogels [15], cellulose nanofiber/ZSM-5 zeolite/polyethyleneimine aerogel [16], chitosan/P(AA-co-DMAEMA) aerogels [17], CNF-TA-PMMT-PEI aerogel [18], alginate/melamine/chitosan aerogel [19], and others. After five or more adsorption–desorption cycles, numerous composite aerogels maintain a high adsorption capacity for heavy metals. Although a large number of modification methods have been applied to enhance the adsorption capacity of nanocellulose, it is still necessary to develop better modification methods to avoid strict reaction conditions and the use of toxic chemicals.

Recently, some catechol compounds, such as polydopamine, have been introduced to NCA to improve adsorption capacity during water purification [20]. Tannic acid (TA) can be extracted easily from plants as a cheap and natural catechol [21]. Due to its abundant catechol groups, TA exhibits a high chelating affinity for many metal ions. However, an immobilization process is needed before TA can be used as a biosorbent for water treatment because of its water solubility. Although some substrates, such as collagen [22], collagen fibers [23], and mesoporous silica [24], have been used for TA fixation, preparing TAs containing high-capacity adsorbents using facile and inexpensive methods remains challenging.

Although there have been some reports on TA and nanocellulose composite gel materials at present, TA can act as a cross-linking agent to stabilize the network structure and endow the aerogel with certain functions, such as antibacterial and antioxidant properties. However, the TA composite aerogel based on nanocellulose is still relatively lacking in the research of heavy metal adsorption, and more attempts are needed to better illustrate the feasibility of TA composite aerogel in the removal of heavy metal ions. By investigating the adsorption performance of TA and nanocellulose composite aerogel on heavy metal ions, this study provides a new idea for the preparation of cellulose-based aerogel with excellent adsorption performance. In this work, a porous aerogel adsorbent based on cellulose nanofibrils (CNFs), TA, and polyvinyl alcohol (PVA) was successfully prepared using a one-pot method. This cellulose nanofibril/tannic acid/polyvinyl alcohol (CNF/TA/PVA) aerogel absorbent was characterized by FT-IR, SEM, and TGA. Then, its simulated ability to remove Cd^2+^, Pb^2+^, and Cu^2+^ and the adsorption processes for each heavy metal were systematically studied.

## 2. Materials and Methods

### 2.1. Materials

Nanocellulose was purchased from North Century Cellulose Material Co., Ltd. (Xuzhou, China). The standard solutions, including Cu^2+^, Pb^2+^, and Cd^2+^ (in Cu(NO_3_)_2_, Pb(NO_3_)_2_, and Cd(NO_3_)_2,_ 1 mol/mL, respectively), were purchased from the National Nonferrous Metals and Electronic Materials Analysis and Testing Center National Standard (Beijing) Inspection and Certification Co., Ltd. (Beijing, China). Tannic acid (TA), polyvinyl alcohol (PVA), and ethanol were purchased from Shanghai Maclin Biochemical Technology Co., Ltd. (Shanghai, China).

### 2.2. Preparation of the CNF/TA/PVA Hybrid Aerogel

First, the CNF and TA solutions were placed into one mixture and stirred for 20 min; then, various amounts of ethanol and PVA were added to this mixture under stirring. Excess ethanol was removed by heating. The composition of the mixture (100 g) was set as follows: 1.2 wt.% CNFs, 1 wt.% PVA, and 0.1 wt.% TA. This mixed solution was then pre-frozen in liquid nitrogen and placed into a freeze-dryer. The lyophilized nanocellulose aerogel samples were subsequently deposited into a vacuum-drying oven and dried at 120 °C for 24 h.

### 2.3. Characterizations

#### 2.3.1. Fourier Transform Infrared Spectroscopy (FT-IR)

FT-IR spectroscopy was performed using a Fourier transform infrared spectrometer (Shimadzu Corporation, Kyoto, Japan). The spectra have a recording width of 400 to 4000 cm^−1^ with a resolution of 2 cm^−1^.

#### 2.3.2. Scanning Electron Microscopy (SEM)

The surface morphologies of the samples were observed through scanning electron microscopy. The samples were cut into liquid nitrogen with a blade. Then, each cross-sectional sample was sprayed with gold and observed using SEM.

#### 2.3.3. Thermogravimetric Analysis (TGA)

The thermal behavior of the samples was studied via thermogravimetric analysis (TGA) using the German Netzsch TG209F3 Tarsus thermogravimetric analyzer. The temperature was increased from room temperature to 800 °C at a heating rate of 10 °C/min in a nitrogen environment with a flow rate of 20 mL/min.

### 2.4. Adsorption Behaviors of the CNF/TA/PVA Hybrid Aerogel

Quantities of Cu(NO_3_)_2_, Pb(NO_3_)_2_, and Cd(NO_3_)_2_ were dissolved in water to form the solution needed, ranging from 0 to 120 mg/L. To investigate the adsorption properties of the CNF/TA/PVA hybrid aerogel for heavy metal ions, adsorption experiments were conducted with the initial concentration of 100 mg·L^−1^ for Cu^2+^, Pb^2+^ and Cd^2+^, respectively, in the pH range of 2–6. The CNF/TA/PVA hybrid aerogel (0.05 g) was added to a heavy metal ion solution at a constant temperature of 60 °C, and the adsorption time was 4 h. Finally, the residual metal ion concentrations in the solution were determined using atomic absorption spectrophotometry (AAS).

The adsorption principle is the combination of chemical substances and absorbents. The adsorption process is controlled by interactions between the adsorbate and the adsorbent. The relationship between the surface concentration and bulk concentration of the adsorbent at a constant temperature is often described by the adsorption isotherm [24]. The concentration of metal ions in each adsorption experiment was determined using an atomic absorption meter, which was then used for isotherm and kinetic model analysis. The adsorption capacity (*q_e_*) at equilibrium can be calculated using Equation (1):

(1) $$q_{e}=\frac{V\left(c_{o}-C_{e}\right)}{m}$$

where *q_e_* (mg/g) is the equilibrium amount of metal ions adsorbed per mass of the aerogel; *V* (mL) is the volume of the solution; *c_o_* and c*_e_* are the initial and equilibrium metal ions (mg·L^−1^); and *m* (g) is the mass of the adsorbent.

#### 2.4.1. Adsorption Kinetics

Pseudo-first-order models, pseudo-second-order models, and the ion diffusion model are often used to describe the kinetics of adsorption behavior. The pseudo-first-order and pseudo-second-order reaction models and the ion diffusion model are represented by the following linear Equations (2) and (3) and the non-linear Equation (4) [25]:

(2) $$\ln\left(q_{e}-q_{t}\right)\;=\;\ln q_{e}-k_{1}\frac{t}{2.303}$$

(3) $$\frac{t}{q_{t}}\;=\;\frac{1}{k_{2}q_{e}^{2}}+\frac{t}{q_{e}}$$

(4) $$q_{t}\;=\;k_{id}t^{0.5}+c_{i}$$

Here, *q_t_* (mg·g^−1^) is the adsorption capacity at time *t* (min); *k*_1_ (min^−1^) and *k*_2_ (g·mg^−1^·min^−1^) are the rate constants of the pseudo-first-order and pseudo-second-order reaction, respectively; *k_id_* (mg·g^−1^·min^−0.5^) is the rate constant of intra-particle diffusion; and *c_i_* (mg·g^−1^) is the characteristic parameter representing boundary layer thickness.

#### 2.4.2. Adsorption Isotherm

The Langmuir isotherm models are shown as linear Formula (5):

(5) $$\frac{c_{e}}{q_{e}}=\frac{c_{e}}{q_{max}}+\frac{1}{K_{L}q_{max}}$$

where *q_max_* is the maximum adsorption capacity (mg/g); *c_e_* is the equilibrium concentration of free adsorbent molecules (mg/L); and *K_L_* (L/mg) is the Langmuir constant related to the free energy of adsorption. In particular, *K_L_* is an important parameter for predicting the affinity between the adsorbate and the adsorbent. This factor is used to calculate an *R_L_* called a dimensionless equilibrium parameter, the value of which provides significant information about the adsorption strength, as given by the following equation:

(6) $$R_{L}=\frac{1}{1+K_{L}C_{O}}\;$$

The Freundlich model is an empirical equation that describes the adsorbent’s behavior on heterogeneous surfaces at active sites with non-uniform energy. The equation of this model is expressed via the following non-linear Equations (6) and (7) [26]:

(7) $$\ln q_{e}\;=\;\ln K_{F}+\frac{1}{n}\ln c_{e}\;$$

(8) $$q_{e}\;=\;K_{F}c_{e}^{1/n}$$

where *K_F_* is the Freundlich capacity constant and *n* is the adsorption strength.

## 3. Results and Discussion

### 3.1. Preparation and Characterizations of Aerogels

**Figure 1:**
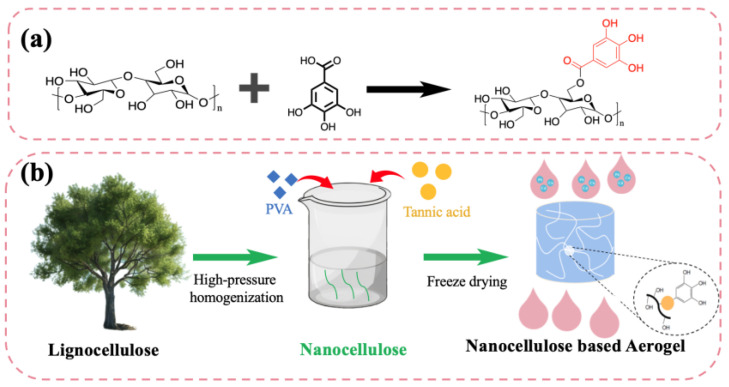
A schematic diagram showing the preparation of the CNF/TA/PVA microporous aerogel: (**a**) the reaction mechanism of CNFs and TA and (**b**) the preparation process of the CNF/TA/PVA hybrid aerogel.

The process of preparing nanocellulose-based aerogels from lignocellulose is shown in Figure 1. TA molecules are rich in multiple phenolic hydroxyl groups, which can form stable complexes with hydroxyl groups on nanocellulose chains through hydrogen bonding or esterification reactions, thereby enhancing the structural stability and functionalization characteristics of the material (Figure 1a). Natural lignocellulose is subjected to acid hydrolysis with sulfuric acid to obtain uniformly sized and well-dispersed nanocellulose. Then, TA from natural plant sources is introduced for surface functionalization. Finally, an NCA with a porous structure is constructed through freeze-drying (Figure 1b). This aerogel material has advantages such as a light weight, high porosity, and specific surface area; it also exhibits excellent adsorption performance for heavy metals (Pb, Cu, and Cd) due to the introduction of TA and can be widely applied in water pollution. The entire process is environmentally friendly and mild in condition and uses a wide range of raw material sources, providing a theoretical basis and practical approach for the development of sustainable materials and the design of environmentally friendly functional materials.

**Figure 2:**
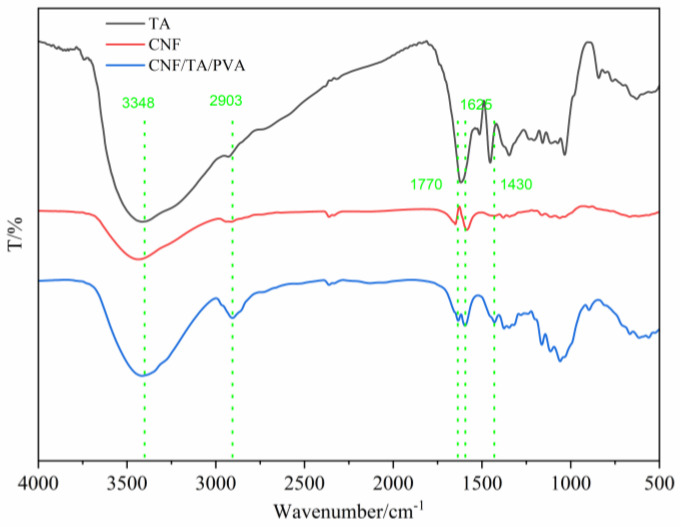
FT-IR spectra of CNF and TA aerogels and the CNF/TA/PVA hybrid aerogel.

The FT-IR spectra of CNF and TA aerogels and the CNF/TA/PVA hybrid aerogel are shown in Figure 2. The absorption peak at 3348 cm^−1^ corresponds to the stretching vibration of -OH, and indicates the presence of abundant hydroxyl groups in the prepared hybrid aerogel. The interactions between hydroxyl groups through hydrogen bonds have a significant impact on the aggregated structure and properties of nanocellulose. The peak at 2903 cm^−1^ is attributed to the stretching vibration of C-H, reflecting the presence of saturated C-H structures on the cellulose molecular chain. The absorption peak at 1664 cm^−1^ is caused by the stretching vibration of C-C and the in-plane bending vibration of -OH, confirming a skeleton structure of cellulose molecules. The absorption peak at 1430 cm^−1^ corresponds to the bending vibration of -CH_2_, which is characteristic of the side-chain structure of cellulose molecules. These cellulose characteristic peaks are highly consistent with previous research results, indicating that the basic chemical structure of cellulose is completely retained [27].

The C=O group absorption peak at 1700 cm^−1^ in the CNF/TA/PVA hybrid aerogel is due to the presence of ester carbonyl groups, indicating the introduction of functional groups or compounds containing ester carbonyl groups. The absorption peaks in the 1240–1313 cm^−1^ band are typical characteristics of C-O-C groups, indicating that TA was successfully grafted onto the nanocellulose skeleton structure. The formation of C-O-C bonds establishes a chemical connection between CNFs and TA, which not only alters the surface chemical properties of the prepared hybrid aerogel but may also have a significant impact on its physical properties, including hydrophilicity, mechanical properties, etc.

To further verify the curing degree of TA onto the nanocellulose skeleton, the prepared CNF/TA/PVA hybrid aerogel was placed in water and shaken at 100 rpm for 60 min in a shaker. Subsequently, the supernatant was detected under ultraviolet light at 275 nm, and no absorption peak was found for TA; this suggests that TA was completely cured onto the nanocellulose skeleton model, with none lost to the solution during the shaking process. This proves that the prepared composite system has well-founded stability and structural integrity.

**Figure 3:**
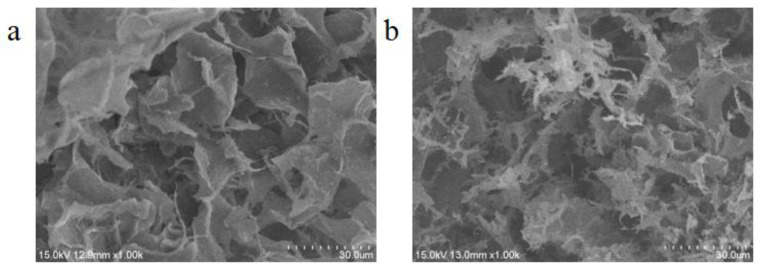
SEM images of the CNF aerogel (**a**) and the CNF/TA/PVA hybrid aerogel (**b**).

SEM is one of the most important testing methods for characterizing the microstructure of material surfaces; it can visually display the pores, surface morphologies, and structural features of materials. Figure 3 shows the SEM images of the CNF aerogel and the CNF/TA/PVA hybrid aerogel, showing significant differences in their microstructure. The CNF aerogel presents a distinct continuous pore structure with a uniform surface and relatively dispersed pores (Figure 3a). This network structure indicates that the CNF aerogel has valid structural stability and a large specific surface area, conducive to its high performance in applications such as adsorption and energy storage. Compared with the CNF aerogel, the porosity of the CNF/TA/PVA hybrid aerogel (Figure 3b) significantly increased; the pore size tended to decrease while more connection structures emerged simultaneously. These changes indicate that the introduction of TA and PVA enhances the physical and chemical cross-linking between the CNF aerogel networks.

Specifically, TA, as a natural polymer compound, has comprehensive complexing properties and can form hydrogen bonds and electrostatic interactions with CNFs. PVA plays a further cross-linking role in the aerogel, enhancing its mechanical strength and stability. Due to the introduction of TA and PVA, the cross-linking degree of the CNF/TA/PVA hybrid aerogel significantly improved. This structural optimization enhances the mechanical properties of the material and greatly improves its efficiency in adsorbing heavy metal ions. Enhanced cross-linked networks can provide more adsorption sites, effectively improving the adsorption capacity of the aerogel for pollutants. This result indicates that the CNF/TA/PVA hybrid aerogel has great potential for environmental governance and removing pollutants.

The thermal stability of aerogels was studied using TGA in a nitrogen atmosphere. The weight loss of the samples below 150 °C was attributed to the removal of physically and chemically combined water [28].

**Figure 4:**
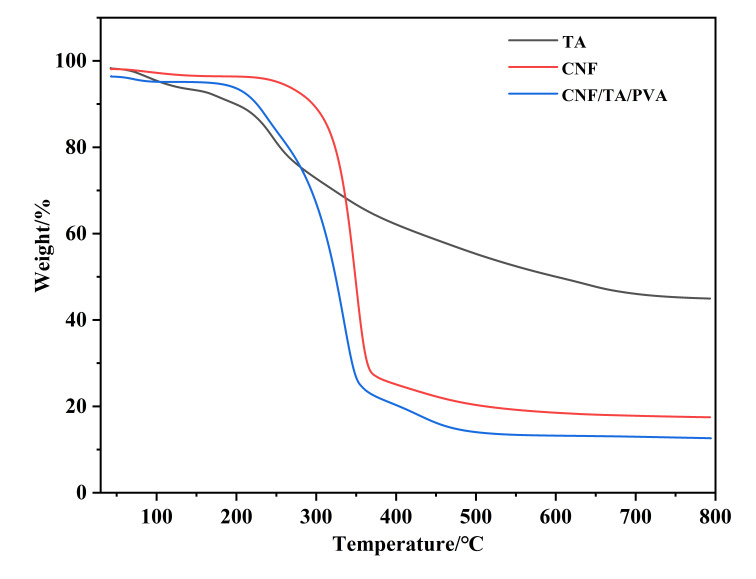
The TGA of the CNF and TA aerogels and the CNF/TA/PVA hybrid aerogel.

Although the samples were freeze-dried before testing, it was difficult to remove all the bonded water molecules due to hydrogen interactions. The initial and maximum pyrolysis temperatures of the CNF aerogel were 220.5 °C and 358.5 °C, which is typical for cellulose degradation behavior and closely related to the sulfate-activated dehydration reaction [29]. These temperatures were 207.9 °C and 337.7 °C for the CNF/TA/PVA hybrid aerogel, among which the initial degradation temperature of the TA-containing nanocellulose aerogel was slightly lower than that of the CNF aerogel. Although this temperature was slightly lower for the modified aerogel than that of the CNF aerogel, it still expressed high thermal stability, similar to previous studies [30,31] (Figure 4).

### 3.2. Adsorption Behavior of Aerogels

**Figure 5:**
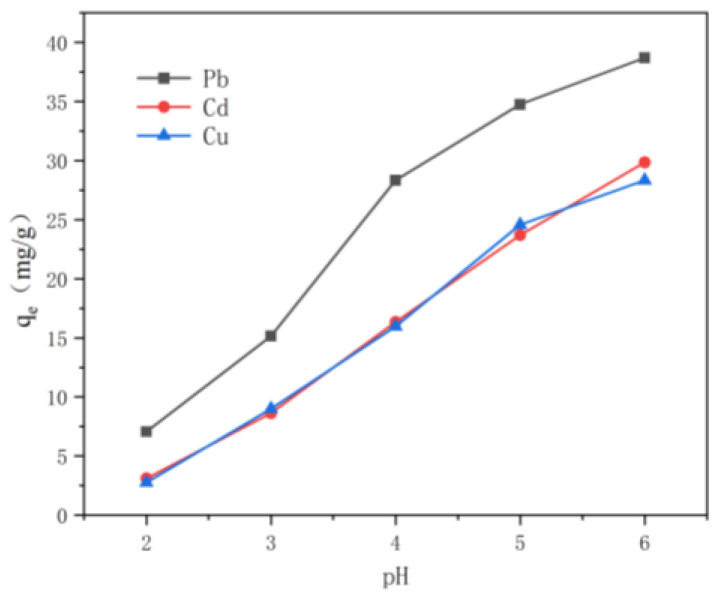
The influence of pH on the removal efficiencies of Cu^2+^, Pb^2+^, and Cd^2+^.

The adsorption site and the physicochemical state of heavy metal ions are important factors in the adsorption capacity of adsorbents. They can be affected by pH and the initial concentration of the solution. To investigate the effect of pH, the adsorption capacity of the CNF/TA/PVA hybrid aerogel to Cu^2+^, Cd^2+^, and Pb^2+^ was studied, as shown in Figure 5. Since the precipitation of these heavy metal ions occurs above the pH range of 7.0–7.5 in aqueous solutions [27], the pH of the solution was set at a range of 2 to 6 in this study. While the adsorption capacity of the aerogel on the three metal ions is highly dependent on the solution’s pH, that of the CNF/TA/PVA hybrid aerogel for metal ions increases with rising pH levels. Specifically, in the pH range of 2.0 to 6.0, the adsorption capacities increased from 7.40 to 38.2 mg/g for Pb^2+^, 3.35 to 30.0 mg/g for Cd^2+^, and 2.90 to 28.3 mg/g for Cu^2+^. The increased ionization of hydroxyl groups in tannic acid and the aerogel with rising pH levels is a key reason for the prepared aerogel’s enhanced adsorption capacity. The maximum adsorption capacity was pH 6. Therefore, the following adsorption experiments were carried out at pH 6.

**Figure 6:**
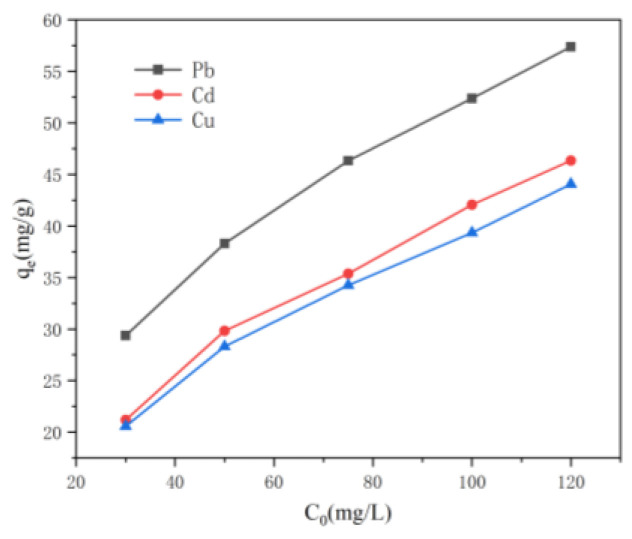
The influence of the initial concentration on the removal efficiencies of Cu^2+^, Pb^2+^, and Cd^2+^.

Figure 6 shows the effect of the initial concentration on the adsorption capacity of the CNF/TA/PVA hybrid aerogel in response to Pb^2+^, Cd^2+^, and Cu^2+^. The results show that the adsorption capacity increased when increasing the initial concentration, and the maximum adsorption of this prepared aerogel in response to different metal ions was Pb^2+^ > Cd^2+^ ≈ Cu^2+^; this indicates that the sample had the highest adsorption affinity for Pb^2+^. The adsorption performance of the CNF/TA/PVA hybrid aerogel for Pb^2+^, Cd^2+^, and Cu^2+^ was attributed to this prepared aerogel’s pore structure and the introduction of TA. Hydroxyl and carbonyl play a vital part in removing heavy metals. The adsorption mechanisms of this hybrid aerogel on Pb^2+^, Cd^2+^, and Cu^2+^ are mainly chelation, coordination, and ion exchange [32,33].

### 3.3. Adsorption Mechanism of the CNF/TA/PVA Hybrid Aerogel

Adsorption kinetics describe the adsorption rate and its mechanism. The experimental results are fitted to a pseudo-first-order kinetic model and a pseudo-second-order kinetic model. The pseudo-first-order kinetic model is based on the relationship between the adsorption rate and the number of unoccupied sites. In this model, the occupancy rate of adsorption sites is proportional to the number of unoccupied adsorption sites. In the pseudo-second-order kinetic model, the adsorption rate is related to the square of the product detailing the number of unoccupied and occupied positions.

**Figure 7:**
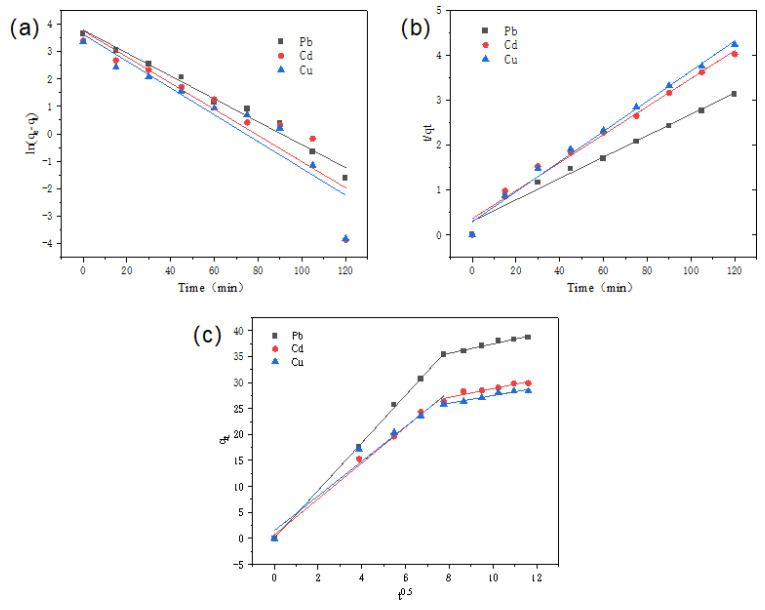
Fitting curves of pseudo-first-order kinetic (**a**), pseudo-second-order kinetic (**b**), and ion diffusion (**c**) models.

The fitting correlation coefficients R^2^ of the pseudo-second-order kinetic model are 0.9962, 0.9807, and 0.9891, which are higher than those of the pseudo-first-order kinetic model (0.9722, 0.8146, and 0.8238) (Table 1 and Figure 7). This indicates that adsorption kinetics are more consistent with the pseudo-second-order kinetic model. The calculated equilibrium adsorption values (q_e_, cal) using the pseudo-second-order kinetic model agree well with the experimental results (q_e_, exp). This is similar to the results of TA-related adsorption materials reported by Sun et al. [22], indicating that chemisorption is the limiting step during the adsorption process. The surface functional groups of CNF/TA/PVA undergo a chemisorption reaction with heavy metal ions.

**Figure 8:**
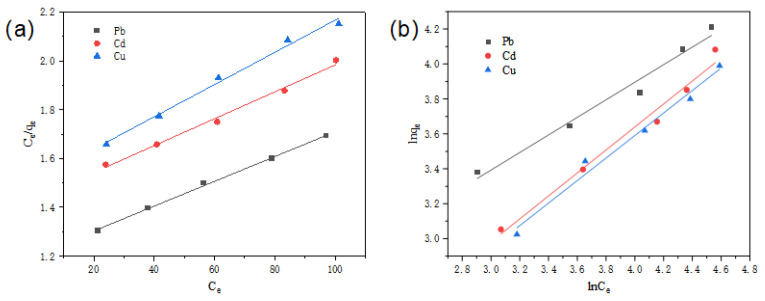
Adsorption isotherm fitting curves of Langmuir (**a**) and of Freundlich (**b**).

To further explore the adsorption process of aerogels in response to heavy metal ions, the Langmuir and Freundlich adsorption isotherm models were used to fit the results and clarify the interaction behavior between the adsorbates and the adsorbents. While the Langmuir isotherm model is based on monolayer adsorption with uniformly distributed adsorption sites on a homogeneous surface, the Freundlich isotherm model is used to understand adsorption on heterogeneous surfaces with multiple adsorption layers [34]. The R^2^ values of the Langmuir fitted adsorption of Pb^2+^, Cd^2+,^ and Cu^2+^ were 0.998, 0.992, and 0.991, respectively, which are higher than those of the Freundlich fitted adsorption R^2^ values (0.976, 0.981, and 0.983), as shown in Table 2 and Figure 8. This indicates that the Langmuir model provides a good fit and can better describe the adsorption process of aerogels, proving that it involves monolayer adsorption. In addition, the isothermal model parameters calculated in Table 2 (0 < R_L_ < 1) indicate that the adsorption process of the CNF/TA/PVA hybrid aerogel is more likely to occur [35]. Moreover, the maximum saturated adsorption capacities of the CNF/TA/PVA hybrid aerogel for Pb^2+^, Cd^2+^, and Cu^2+^ reach 196.850 mg/g, 181.488 mg/g, and 151.515 mg/g, respectively.

### 3.4. Comparison of Adsorption Properties for Different Adsorbents

Table 3 shows a comparison of the adsorption capacity of several nanocellulose-based adsorbents. The TA-modified nanocellulose aerogel prepared in this work reaches a higher level than other cellulose-based aerogels, indicating that the CNF/TA/PVA hybrid aerogel is an excellent adsorbent for heavy metal ions.

### 3.5. Studying the Regeneration of the Adsorbent

In addition to adsorption capacity, reusability is also important for biological adsorbents. In this study, the CNF/TA/PVA hybrid aerogel were treated with EDTA as the eluent through three adsorption–desorption cycles. After three regenerations, the adsorption efficiency of the CNF/TA/PVA hybrid aerogel for Pb^2+^, Cd^2+^, and Cu^2+^ in the new adsorption solution remained above 90% (Table 4), indicating that the CNF/TA/PVA hybrid aerogel is reusable. The incomplete desorption of the adsorbent may be a reason behind the reduced adsorption capacity of the regenerated aerogel; however, this requires further study. Additional research revealed that the CNF/TA/PVA hybrid aerogel exhibit commendable wet strength in aqueous environments, enabling them to undergo three complete adsorption–desorption cycles.

## 4. Conclusions

In this work, a porous aerogel was prepared through physical and chemical cross-linking based on cellulose nanofibrils (CNFs) as the main framework, TA as the adsorbent, and polyvinyl alcohol (PVA) as the reinforcing agent. The prepared CNF/TA/PVA hybrid aerogel exhibited low density, a large specific surface area, and good thermal stability. When the initial adsorption concentration was 100 mg/L, the maximum saturated adsorption capacities of Cd^2+^, Pb^2+^, and Cu^2+^ reached 197 mg/g, 181 mg/g, and 152 mg/g, respectively, demonstrating reasonable adsorption properties. The CNF/TA/PVA hybrid aerogel’s adsorption kinetics conform to the pseudo-second-order kinetic model, and the adsorption isotherms agree with the Langmuir isotherm model. Compared with other adsorption materials and TA-modified materials, the modified CNF/TA/PVA hybrid aerogel in this paper demonstrated excellent adsorption efficiency and reusability and is expected to have excellent application potential in the treatment of heavy metal-containing wastewater.

## Figures and Tables

**Table 1:** Adsorption kinetic fitting parameters.

Metal Ions		Pb^2+^	Cd^2+^	Cu^2+^
Pseudo-first-order kinetic model	R^2^	0.9722	0.8146	0.8238
	K_1_	0.0374	0.0477	0.0517
	q_e, cal_ (mg/g)	36.432	22.781	16.415
Pseudo-second-order kinetic model	R^2^	0.996	0.981	0.989
	K_2_	0.304	0.360	0.290
	q_e, cal_ (mg/g)	38.0	29.2	27.8
Ion diffusion model	R_1_^2^	0.999	0.992	0.970
	K_id1_	4.576	3.451	3.336
	C_1_	0.075	0.697	1.489
	R_2_^2^	0.979	0.913	0.953
	K_id2_	0.911	0.843	0.725
	C_2_	28.301	20.394	20.2
	q_e, exp_ (mg/g)	52.4	42.1	39.3

**Table 2:** Adsorption isotherm fitting parameters.

Metal Ions		Pb^2+^	Cd^2+^	Cu^2+^
Langmuir	R^2^	0.998	0.992	0.991
	K_L_	0.005	0.006	0.007
	R_L_	0.800	0.769	0.741
	q_max_ (mg/g)	197	181	152
Freundlich	R^2^	0.976	0.981	0.983
	n	1.99	1.520	1.55
	K_F_	6.573	2.737	2.748

**Table 3:** A comparison of the adsorption properties of different adsorbents.

Materials	Metal Ions	Adsorbent Concentration	Adsorption Capacity (mg/g)	Ref.
Corn stalk-grafted cellulose (AGCS-Cell)	Cd^2+^	100 mg/L	21.4	[36]
Carboxylated cellulose (Cell-EDTA)	Cd^2+^	100 ppm	33.2	[37]
	Pb^2+^		41.2	
Schiff base cellulose (Gu-Mc)	Cu^2+^	100 mg/L	83.0	[38]
	Cd^2+^		68.0	
	Pb^2+^		52.0	
Multifunctionalized cellulose (TMCS) Carbon nanotube	Cd^2+^	200 mg/L	54.7	[39]
Tannin/cellulose microspheres	Pb^2+^	100 mg/L	23.8	[40]
Tannin/nanocellulose composite	Cu^2+^	100 mg/L	46.1	[25]
	Pb^2+^		50.0	
	Cr^6+^		103	
TA@CNF–cardanol-derived siloxane	Cu^2+^	100 mg/L	47.6	[41]
CNF/TA/PVA hybrid aerogel	Pb^2+^	100 mg/L	62.4	This work
	Cd^2+^		52.1	
	Cu^2+^		49.3	

**Table 4:** The reusability of the CNF/TA/PVA hybrid aerogel.

Heavy Metal Ions	Adsorption Capacity (mg/g)	Adsorption Efficiency of Reused Aerogels (%)		
		1	2	3
Pb^2+^	62.4	95.2	93.2	90.5
Cd^2+^	52.1	96.6	94.7	91.8
Cu^2+^	49.3	98.4	97.4	97.2

## Data Availability

Data is contained within the article.

## Abbreviations

NCAs	Nanocellulose-based composite aerogels
CNFs	Cellulose nanofibrils
TA	Tannic acid
PVA	Polyvinyl alcohol
CNF/TA/PVA	Cellulose nanofibril/tannic acid/polyvinyl alcohol hybrid
